# Frequency and Clinical Features of *Candida* Bloodstream Infection Originating in the Urinary Tract

**DOI:** 10.3390/jof8020123

**Published:** 2022-01-27

**Authors:** Meital Elbaz, Amanda Chikly, Ronnie Meilik, Ronen Ben-Ami

**Affiliations:** 1 Infectious Disease Unit, Tel Aviv Sourasky Medical Center, Tel Aviv 64239, Israel; meitale@tlvmc.gov.il (M.E.); amandac@tlvmc.gov.il (A.C.); 2 Sackler Faculty of Medicine, Tel Aviv University, Tel Aviv 69978, Israel; ronniemeilik@gmail.com

**Keywords:** candidemia, blood stream infection, urinary tract infection

## Abstract

The urinary tract is considered an uncommon source of *Candida* bloodstream infection (CBSI). We aimed to determine the source of CBSI in hospitalized patients, and to compare clinical and microbiological features of CBSI originating in the urinary tract (U-CBSI) and non-urinary CBSI (NU-CBSI). Of 134 patients with CBSI, 28 (20.8%) met criteria for U-CBSI, 34 (25.3%) had vascular catheter-related CBSI and 21 (15.6%) had a gastrointestinal origin. Compared to NU-CBSI patients, patients with U-CBSI were older with higher rates of dementia. Bladder catheterization for urinary retention and insertion of ureteral stents or nephrostomies were risk factors for U-CBSI. Fifty percent of U-CBSI cases occurred within 48 h of hospital admission, versus 16.9% of NU-CBSI (*p* < 0.0001). The mortality rate was lowest for CBSI originating in the urinary tract and highest for CBSI of undetermined origin. CBSI of undetermined origin remained associated with higher mortality in a Cox regression model that included age, *Candida* species, Pitt bacteremia score and neutropenia as explanatory variables. U-CBSI may be increasing in frequency, reflecting extensive use of bladder catheters and urologic procedures in elderly debilitated patients. Distinct clinical features are relevant to the diagnosis, treatment and prevention of U-CBSI.

## 1. Introduction

*Candida* species are among the most frequent causes of bloodstream infections in hospitalized patients [1,2,3,4], and are associated with the highest case-fatality rate of all common nosocomial pathogens [1,5]. While the annual incidence and fatality rate have decreased over the past decade, candidemia remains an important cause of morbidity, mortality and healthcare expenditure [6,7].

*Candida* bloodstream infection (CBSI) is thought to arise endogenously from the gastrointestinal tract, with the skin being a less frequent source of infection [8]. *Candida* spp. frequently colonize the skin and mucosal surfaces, and factors contributing to dysbiosis and fungal overgrowth, breaches in the integrity of the mucosal barrier or skin, and impaired innate immunity may lead to fungal invasion of the bloodstream and sepsis [2]. Well characterized risk factors include exposure to broad spectrum antibiotics, gastrointestinal surgery, chemotherapy-induced mucositis, central venous catheterization and neutropenia [2].

While *Candida* spp. are frequently isolated from the urine of hospitalized patients, the urinary tract is regarded as an infrequent source of CBSI in adults [9]. Patients with candiduria typically have multiple comorbidities, notably diabetes mellitus, malignancy and recent antibiotic treatment, and most have indwelling bladder catheters [9,10]. Most cases of candiduria clear spontaneously or after removal of a bladder catheter [9]. In longitudinal studies, the frequency of candidemia among patients with candiduria ranged from 1.3% in a general hospital population [9] to 8% in ICU patients [11]. Ascending infection and candidemia are usually associated with urinary tract obstruction and stasis [9,10,12,13].

Nosocomial candiduria is increasing in frequency [14], with some centers now reporting *Candida* spp. as the most frequent pathogens cultured from the urine of critically ill patients [15]. This trend, driven by extensive use of bladder catheters in elderly debilitated patients [16], together with improved prevention of vascular catheter-related bloodstream infections [7], may increase the importance of the urinary tract as a source of candidemia. In this study, we aimed to determine the frequency of the urinary tract as a source of candidemia, and to compare risk factors, clinical features and outcomes between candidemia of urinary and non-urinary origin.

## 2. Materials and Methods

### 2.1. Study Design and Population

This was a retrospective cohort study of adult patients with microbiologically diagnosed *Candida* bloodstream infection, admitted to the Tel Aviv Sourasky Medical Center (TASMC), a tertiary-level academic hospital in Tel Aviv, Israel. The aim was to compare risk factors, clinical features and outcomes between patients with urinary source (U-) CBSI and non-urinary source (NU-) CBSI. We included hospitalized adults (≥18 years) with CBSI, defined as at least one blood culture growing *Candida* spp., admitted from January 2017 to December 2020. Patients meeting study inclusion criteria were identified by querying the computerized microbiology laboratory databases.

We defined U-CBSI based on criteria proposed by Cuervo et al. [17], with modification: An episode of candidemia fulfilling at least two of the following criteria, concomitant with the onset of candidemia: 1. Signs and symptoms of upper urinary tract infection (flank pain and tenderness); 2. transurethral instrumentation; 3. recovery of the same *Candida* species from blood and urine cultures obtained within 24 h of the onset of candidemia; and 4. radiological (ultrasound or computed tomography) findings supporting UTI (abscess, evidence of pyelonephritis, etc.); and no alternative explanation for CBSI. The microbiological criterion (criterion 3) was not a requirement. We therefore defined U-CBSI as probable if the same *Candida* species was recovered from urine and blood, and presumptive if this criterion was not met. Cases of NU-CBSI were further classified as candidemia originating from the gastrointestinal or hepatobiliary tract, central vascular catheter associated candidemia and candidemia of undetermined origin (Table S1). All cases were reviewed by two study investigators (RB and ME), and their classification was agreed upon based on microbiological and clinical data.

Clinical outcomes compared between groups were 30 day mortality, length of hospitalization, presence of metastatic *Candida* infection (endophthalmitis, endocarditis or infection of other organs or sterile sites), hemodynamic shock and need for vasopressor treatment, need for mechanical ventilation, acute kidney injury (assessed using RIFLE criteria [18]), duration of fever ≥38.0 °C and relapsed infection. Relapsed infection was defined as a second episode of candidemia that occurred at least 7 days after the last positive blood culture positive for *Candida* spp. and within 90 days of the first candidemia episode.

This study was reviewed and approved by the TASMC ethics committee (approval no. 0059-18-TLV). Requirement for informed consent was waived considering the retrospective observational nature of the study.

### 2.2. Data Collection

Data were retrieved from the hospital electronic medical record system and laboratory computerized database. Collected covariates included demographic data, comorbidities (quantified using the Charlson comorbidity score [19]), general risk factors for CBSI (central vascular catheterization, parenteral nutrition, hemodialysis, abdominal surgery, gastrointestinal leak, neutropenia, ICU stay, chemotherapy and other immunosuppressive therapy) and specific risk factors for urogenital candidiasis (urinary *Candida* colonization, urinary tract obstruction, bladder catheterization and other urologic procedures). Severity of sepsis was assessed using the Pitt bacteremia score [20].

CBSI was defined as hospital-acquired when the index culture was collected >48 h after admission. Presence of metastatic infection, including ocular candidiasis and endocarditis, was determined from medical records. Endocarditis was defined according to Dukes criteria [21].

### 2.3. Microbiological Methods

Blood culture bottles were incubated in the BacT/Alert Virtuo system (bioMerieux, Marcy L’Etoile, France) for up to 5 days. Culture time-to-positivity was determined and recorded by the BacT/Alert system. Semi-quantitative urine cultures were performed using the Diaslide device (Novamed, Jerusalem, Israel), according to the manufacturer’s instructions. Colony densities consistent with ≥10^3^ CFU/mL were reported as positive. Semiquantitative culture of extracted central vascular catheter tips was performed using the plate-roll method [22]. The tip was considered positive if 15 or more colonies were detected after overnight incubation on blood agar. *Candida* species were identified using growth on CHROMagar *Candida* (CHROMagar, Paris, France), the Vitek 2 system with the ID-YST card (bioMerieux) and VITEK MS (bioMerieux). Antifungal susceptibility testing was performed using Vitek 2 and Clinical and Laboratory Standards Institute breakpoints [23]. Concomitant bacterial bloodstream infection was defined as the presence of pathogenic bacteria in blood culture taken 72 h before or after index *Candida* culture. Non-recurring growth in blood culture of bacteria included on the NHSN list of common commensals [24] was considered a contaminant.

### 2.4. Statistical Analyses

Patient, disease and treatment variables were described within each patient cohort (U-CBSI and NU-CBSI), using number (percentage) for categorical variables, and value (interquartile range (IQR)) for continuous variables. Between-group differences were assessed using Fisher’s exact test for categorical variables, and Student’s *t*-test or the Wilcoxon rank sum test for normally and non-normally distributed continuous variables, respectively.

Survival analyses were performed by plotting Kaplan–Meier curves and using the log-rank test to determine the effect of source of candidemia and other covariates on survival curves. Variables found to be significantly associated with all-cause mortality (*p* < 0.1) were further assessed using Cox proportional hazards modeling. The proportional hazards assumption was checked for each model by testing the time-dependence of covariates and by assessing Schoenfeld residuals after model fitting.

A type I error of <0.05 was considered statistically significant. Calculations were done in Stata 15.0 (Statacorp, College Station, TX, USA).

## 3. Results

The study cohort included 134 patients with CBSI (median age was 71 years, range, 18–95 years, 53 (39.5%) females and 81 (60.4%) males). Twenty-eight patients (20.8%) met the criteria for U-CBSI; 23 patients (17.1%) had probable U-CBSI and 5 (3.7%) had presumptive U-CBSI (Tables S1 and S2). Among the 106 patients with NU-CBSI, 55 (41.7%) had an identifiable source of candidemia: 34 (32.0%) with vascular catheter-related candidemia, 14 (13.2%) with gastrointestinal origin and 7 (6.6%) with hepatobiliary origin of candidemia. Neutropenia was present in 16 patients (11.9%) (Table 1 and Table S2).

Patients with U-CBSI were older than patients with NU-CBSI (median age, 80 years versus 69.5 years, *p* = 0.011) and had higher rates of dementia (42.8% versus 16.0%, *p* = 0.004). Compared to patients with NU-CBSI, patients with U-CBSI were more likely to be hospitalized in medicine wards and less likely to be in intensive care units or the hematology department (*p* = 0.004) (Table 1). Candidemia occurred earlier in the hospital stay for U-CBSI compared to NU-CBSI (median time from admission, 2.5 days versus 11 days, respectively; *p* = 0.0002). CBSI was detected within 2 days of hospital admission in 14 patients U-CBSI (50%) and 18 patients (16.9%) with NU-CBSI (*p* < 0.0001). Most cases of U-CBSI on admission were observed in patients admitted with urinary bladder outlet obstruction and treated with insertion of a bladder catheter or exchange of an obstructed catheter (Table S2).

Most known risk factors for invasive candidiasis, including abdominal surgery, gastrointestinal leak, intraabdominal infection, enteral feeding, neutropenia, ICU stay and mechanical ventilation, were significantly less frequent in patients with U-CBSI than in those with NU-CBSI (Table 1). Sixteen patients (11.9%) had *Candida* urinary colonization prior to infection, with no difference between groups. Prior urologic surgery was more frequent for U-CBSI than NU-CBSI (28.5% versus 6.6%, respectively; *p* = 0.003). Bladder catheter was present at the time of infection in 93 patients (69.4%), with no significant difference between groups. However, bladder catheterization for urinary retention was significantly more frequent for U-CBSI (odds ratio (OR) 5.0, *p* = 0.002), whereas bladder catheter insertion for monitoring urinary output was more frequent for NU-CBSI (OR 0.6, *p* = 0.033). Nephrostomy tubes and ureteral stents were significantly more frequent for patients with U-CBSI (OR 13.2, *p* < 0.0001 and OR 4.5, *p* = 0.011, respectively; Table 1).

The severity of sepsis, assessed using the Pitt bacteremia score, was greater for NU-CBSI than for U-CBSI (median score, 3 versus 1, *p* = 0.014). Patients with U-CBSI were more likely to be febrile than were patients with NU-CBSI (67.8% versus 41.5%, *p* = 0.019). Other clinical features, including proportion of patients with hemodynamic shock, acute kidney injury and duration of fever, were similar between groups (Table 1).

The main *Candida* species in this cohort were *C. glabrata* (n = 58, 43.3%), *C. albicans* (n = 40, 29.8%), *C. tropicalis* (n = 15, 11.2%), *C. parapsilosis* (n = 11, 8.2%) and *C. krusei* (n = 7, 5.2%) (Table 2). Time to blood culture positivity was similar between groups (Table 1). Twenty-one patients (15.6%) had concurrent bacteremia, which was more frequent for U-CBSI than for NU-CBSI (28.5% versus 12.2%, *p* = 0.044). Fourteen isolates (10.5%) were resistant to fluconazole (*C. krusei*, n = 7; *C. parapsilosis*, n = 4; and one each of *C. albicans*, *C. glabrata* and *C. tropicalis*). None of the isolates were resistant to echinocandins. All fluconazole resistant isolates were from patients with NU-CBSI (13.2% versus 0%; *p* = 0.041).

Primary antifungal treatment consisted of an echinocandin (58 patients, 43.2%), fluconazole (50 patients, 37.3%) and liposomal amphotericin B (3 patients, 2.2%) (Table 1). Twenty-three patients (17.1%) did not receive any antifungal treatment, most due to death before the detection of yeast in blood cultures or decision to provide only palliative care. Use of antifungal drugs was not significantly different for U-CBSI and NU-CBSI.

**Figure 1:**
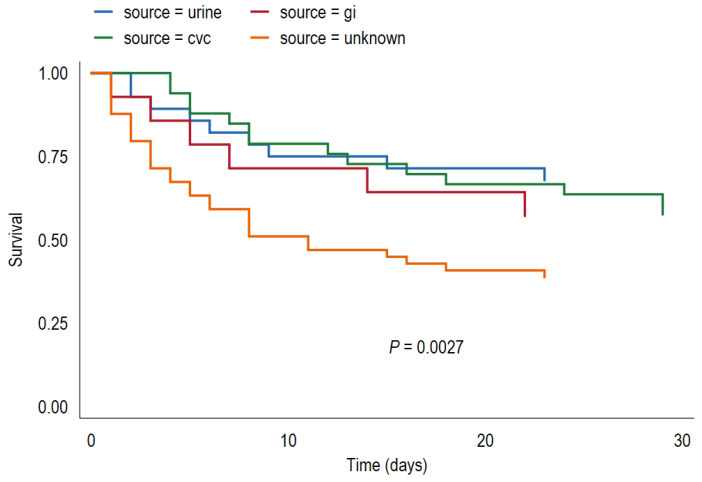
Survival analysis by source of candidemia Kaplan–Meier plot comparing the survival of patients with candidemia originating in the urinary tract (urine), central vascular catheter (cvc), gastrointestinal tract (gi) or an undetermined source (unknown). *p* value was calculated with the log-rank method for the comparison of all groups.

Thirty-day mortality was significantly lower for U-CBSI versus NU-CBSI (32.1% versus 53.7%, log-rank test, *p* = 0.033). Within the NU-CBSI group, 30-day mortality was 44.1%, 47.6% and 62.7% for candidemia associated with vascular catheter, gastrointestinal tract and undetermined source, respectively (Table 3, Figure 1). CBSI of undetermined source remained significantly associated with mortality in a Cox regression survival model, which included as covariates age, Pitt bacteremia score, neutropenia and infection with *C. tropicalis* (Table 4). The mortality rate was not significantly different for primary treatment with echinocandins versus fluconazole for either U-CBSI or NU-CBSI.

The median time to clearance of *Candida* spp. from the bloodstream was 6 days (interquartile range, 4 to 9 days), and was similar between groups. Patients with U-CBSI had significantly shorter hospital stays compared to patients with NU-CBSI (median of 17 days versus 32 days, *p* = 0.007). The rates of metastatic infection (endocarditis and endophthalmitis) were low (6.9% and 2.2%, respectively) with no difference between the groups. Seven patients had relapse of candidemia within 90 days, all of them with NU-CBSI (*p* = 0.3) (Table 3).

## 4. Discussion

In this single-center observational study, the urinary tract was a frequent source of candidemia, accounting for 20.8% of cases, second to vascular catheter-related candidemia, and more frequent than candidemia originating from the gastrointestinal tract. U-CBSI had distinct risk factors and clinical features, and lacked association with many of the well-described risk factors for candidemia, such as vascular catheters, abdominal surgery and ICU stay. Candidemia at the time of hospital admission was strongly associated with U-CBSI, requiring a high level of clinical suspicion to avoid delays in treatment.

The frequency of U-CBSI in this cohort was higher than that reported in other studies that analyzed sources of candidemia. Ang et al. [10] reviewed data from the Mayo clinic, 1985 to 1987, and found that 26 of 249 episodes of candidemia (10.4%) originated in the urinary tract. Cuervo et al. [17] analyzed cases of candidemia from 9 hospitals in Spain and Argentina (2006 to 2015), using case definitions which we adopted and modified; 128 of 2176 episodes of candidemia (5.8%) were classified as originating in the urinary tract. The higher frequency of U-CBSI in the present study may represent regional differences in the utilization of bladder catheters and variable case definitions. It is notable, however, that there are relatively few epidemiological data on the frequency of U-CBSI. For example, clinical studies of invasive candidiasis often do not specifically address the source of candidemia as a relevant variable, and contain no information on procedures involving the urinary tract [25,26,27].

Our findings suggest that the source of candidemia affects both the clinical presentation and outcome of bloodstream infection. Patients with U-CBSI were significantly older and more debilitated than patients with NU-CBSI. However, U-CBSI was associated with significantly lower severity of sepsis, measured using the Pitt bacteremia score. The 30-day mortality rate was lowest for candidemia originating in the urinary tract and highest for candidemia of undetermined origin. Candidemia of undetermined origin was significantly associated with higher mortality in Cox regression survival analysis, possibly due to difficulty in achieving adequate source control in these cases.

Previous studies have found even lower 30-day mortality rates for U-CBSI (14% to 19% [10,17], versus 32.1% in the present study). A potential explanation for this relatively favorable outcome is that U-CBSI is associated with a less exuberant systemic inflammatory response than NU-CBSI, as reflected in the lower Pitt scores of patients with U-CBSI. Similarly, mortality was lower for gram-negative bloodstream infection originating in the urinary tract than in cases associated with other sources, including the peritoneum [28].

Ang et al. [10] reported that U-CBSI is generally brief (median duration, one day) and low-grade (median 1.5 CFU per 10 mL blood). In contrast, we found no difference in the time to blood culture positivity between patients with U-CBSI and NU-CBSI and the median time to blood culture sterilization was six days in both groups. It should be noted, however, that *C. glabrata* was the most frequent causative species of U-CBSI in the present cohort, whereas *C. albicans* accounted for more than 50% of cases in previous studies [10,17]. We hypothesize that *C. glabrata* is associated with a more indolent clinical course than is *C. albicans*, which may result in delayed detection of candidemia and more advanced deep-seated renal involvement. In addition, concurrent bacteremia occurred frequently in this cohort, and might preclude reliable assessment of time to *Candida* culture positivity. An association between *C. tropicalis* and mortality, as seen in this cohort, has been observed in other studies [29], possibly reflecting the intrinsic virulence of this species.

The optimal treatment of U-CBSI has not been defined. Echinocandins have been shown to be superior to fluconazole for the treatment of invasive candidiasis [25,30]. However, pharmacokinetic considerations may favor fluconazole for the treatment of *Candida* urinary tract infections. Echinocandins achieve poor concentrations in urine, but accumulate in the renal parenchyma at high concentrations [31]. In contrast, about 80% of a fluconazole dose is excreted unchanged in the urine [31]. In the present cohort, there was no significant difference in the outcome of U-CBSI treated with echinocandins or fluconazole. Similar findings were reported by Cuervo et al. [17].

Limitations of this study include its single-center setting and small cohort size. The high frequency of U-CBSI observed may reflect local use of bladder catheters and urologic procedures. Further studies are needed to assess whether similar trends are seen in other settings. In addition, drug susceptibility was tested using an automated system (Vitek 2) and not using broth microdilution.

In summary, we found a high proportion of *Candida* bloodstream infections attributable to the urinary tract. U-CBSI was associated with bladder catheterization for urinary retention, urological drainage devices and surgery. Patients with U-CBSI were older and more debilitated than those with NU-CBSI. Patients with these risk factors who have candiduria and signs of sepsis should be evaluated for candidemia, and empirical antifungal treatment should be considered while awaiting results of blood cultures. The source of candidemia is an independent predictor of patient survival and should be considered in epidemiologic studies and clinical trials of invasive candidiasis.

## Figures and Tables

**Table 1:** Characteristics of patients with *Candida* bloodstream infection of urinary and non-urinary origin.

Characteristic	U-CBSI	NU-CBSI	All Patients	*p* Value
	N = 28	N = 106	N = 134	
Age, years	88 (66–88.5)	69.5 (58–81)	71 (59–83)	0.011
Sex				
Male	19 (67.8)	62 (58.4)	81 (60.4)	0.39
Female	9 (32.1)	44 (41.5)	53 (39.5)	
Charlson comorbidity score	3 (1–6)	3 (2–5)	3 (2–5)	0.74
Dementia	12 (42.8)	17 (16.0)	29 (21.6)	0.004
Functional status				
Independent	14 (50.0)	54 (51.4)	68 (51.1)	0.13
Requires help	4 (14.2)	28 (26.6)	32 (24.0)	
Fully dependent	10 (35.7)	23 (21.7)	33 (24.8)	
LTCF residence	6 (27.2)	15 (16.1)	21 (15.67)	0.23
Previous hospitalization (90 days)	11 (39.2)	52 (49.0)	63 (47.01)	0.40
Hospital unit				
Urology	3 (10.7)	2 (1.9)	5 (3.7)	0.004
Internal medicine	19 (67.8)	46 (43.4)	65 (48.5)	
Hematology	0 (0)	18 (16.9)	18 (13.4)	
Surgery	1 (3.5)	7 (6.6)	8 (6)	
ICU	2 (7.1)	25 (23.5)	27 (20.15)	
Source of infection				
Urinary tract ^a^	28 (100)			
Probable	23 (82.1)			
Presumptive	5 (17.8)			
Central venous catheter		34 (32.0)		
Gastrointestinal tract		14 (13.2)		
Hepatobiliary		7 (6.6)		
Undetermined		51 (48.1)		
Presence of bladder catheter	20 (71.4)	73 (68.8)	93 (69.4)	1.0
Acute obstruction	8 (28.5)	6 (5.6)	14 (10.4)	0.002
Monitoring urine output	11 (39.2)	66 (62.2)	77 (57.4)	0.033
Other indication	1 (3.5)	1 (0.9)	2 (1.4)	0.3
Nephrostomy	7 (25.0)	2 (1.9)	9 (6.7)	<0.0001
Ureteral stent	6 (21.4)	5 (4.7)	11 (8.2)	0.011
Ileal conduit	4 (14.2)	5 (4.7)	9 (6.7)	0.091
Long-term bladder catheter	1 (3.5)	6 (5.6)	7 (5.2)	1.0
Time from admission to BSI, days	11 (5–25)	2.5 (0–12.5)	10 (3–23)	0.0002
Time to culture positivity, hours	47.7 (29–71)	38.5 (26–60)	40.5 (28.5–63.5)	0.11
Presence of fever ≥ 38 °C	19 (67.8)	44 (41.5)	63 (47)	0.019
Duration of fever (days)	2.5 (2–3)	1.5 (1–3)	2 (1–3)	0.4
Severity of sepsis ^b^				
Acute kidney injury	7 (25.0)	22 (20.7)	21 (15.6)	0.6
Hypotension	7 (25.0)	44 (41.5)	51 (38.0)	0.12
Duration of hypotension (days)	3 (2–4)	3.5 (2–7)	3 (2–6)	0.3
Pitt score	1 (0–2)	3 (0–6)	2 (0–6)	0.014
Concurrent bacteremia	8 (28.5)	13 (12.2)	21 (15.6)	0.044
Risk factors for candidemia				
Abdominal surgery	1 (3.5)	25 (23.5)	26 (19.4)	0.015
Urologic surgery	8 (28.5)	7 (6.6)	15 (11.2)	0.003
Chemotherapy	5 (18.5)	38 (35.8)	43 (32.3)	0.10
Neutropenia	0 (0)	16 (15.0)	16 (11.9)	0.024
TPN	0 (0)	10 (9.4)	10 (7.5)	0.1
Enteral feeding	6 (21.4)	60 (56.6)	66 (49.3)	0.001
ICU stay	2 (7.1)	37 (34.9)	39 (29.1)	0.004
Mechanical ventilation	3 (10.7)	47 (44.3)	50 (37.3)	0.001
Gastrointestinal leak	0 (0)	14 (13.2)	14 (10.5)	0.041
*Candida* urinary colonization	5 (17.8)	11 (10.3)	16 (11.9)	0.3
Hemodialysis	1 (3.5)	20 (18.8)	21 (15.7)	0.075
Abdominal infection	0 (0)	17 (16.0)	17 (12.7)	0.023
Necrotizing pancreatitis	0 (0)	2 (1.8)	2 (1.5)	1.0
Primary antifungal treatment				
Echinocandin	10 (35.7)	48 (45.2)	58 (43.2)	0.3
Fluconazole	14 (50.0)	36 (33.9)	50 (37.3)	0.1
Liposomal AMB	0 (0)	3 (2.8)	3 (2.2)	1.0
No antifungal treatment	4 (14.2)	19 (17.9)	23 (17.1)	0.7
Time to antifungal treatment (days)	3 (2–4)	2 (1–3)	2 (2–3)	0.081

Categorical variables are presented as number of patients (percent) and continuous variables are presented as median (interquartile range), unless stated otherwise. LTCF: Long term care facility; ICU: Intensive care unit; TPN: Total parenteral nutrition. ^a^ Definitions of probable and presumptive U-CBSI in Methods section and Table S1. ^b^ Assessed during 72 h after the onset of candidemia.

**Table 2:** *Candida* species distribution among urinary and non-urinary candidemia episodes.

Species	U-CBSI	NU-CBSI	All Patients
	N = 28	N = 106	N = 134
*C. albicans*	9 (32.1)	31 (29.2)	40 (29.8)
*C. glabrata*	17 (60.7)	41 (38.6)	58 (43.3)
*C. parapsilosis*	0 (0)	11 (10.3)	11 (8.2)
*C. tropicalis*	2 (7.1)	13 (12.2)	15 (11.2)
*C. krusei*	0 (0)	7 (6.6)	7 (5.2)
*C. lusitaniae*	0 (0)	1 (0.9)	1 (0.75)
*C. dubliniensis*	0 (0)	1 (0.9)	1 (0.75)
Mixed species	0 (0)	1 (0.9)	1 (0.75)

Differences in species distributions among urinary and non-urinary bloodstream infection episodes were not statistically significant (*p* = 0.3).

**Table 3:** Outcomes of urinary and non-urinary source candidemia.

Characteristic	U-CBSI	NU-CBSI	All Patients	*p* Value
	N = 28	N = 106	N = 134	
30-day mortality	9 (32.1)	57 (53.7)	66 (49.2)	0.033
Metastatic infection ^a^				
Endocarditis	0/16 (0)	3/27 (11.1)	3/43 (6.9)	0.2
Endophthalmitis	1/15 (6.67)	0/30 (0)	1/45 (2.2)	0.3
Time to first sterile blood culture, days, median (IQR)	6 (3–7)	3 (2–6)	6 (2–6)	0.9
Length of hospitalization, days, median (IQR)	17 (9–30)	32 (15–62)	25.5 (13–53)	0.007
Relapse of candidemia in 90 days	0 (0)	7 (6.6)	7 (5.2)	0.3

All values represent number of cases (percent within group), unless stated otherwise. ^a^ Percentage calculated out of number of patients who underwent echocardiography and ophthalmoscopy.

**Table 4:** Univariate survival analyses and Cox regression.

Variable	Hazard Ratio	95% Confidence Interval	*p* Value
Univariate			
Age (years)	1.02	1.007–1.037	0.003
Pitt score	1.16	1.10–1.23	<0.0001
Source of candidemia			
Urinary tract	0.53	0.29–0.96	0.039
CVC	0.78	0.47–1.30	0.35
Gastrointestinal	0.90	0.48–1.66	0.73
Undetermined	2.40	1.54–3.71	<0.0001
Neutropenia (previous 30 days)	2.13	1.15–3.94	0.016
Primary antifungal therapy			
Echinocandin	0.69	0.45–1.08	0.10
Fluconazole	0.71	0.45–1.12	0.15
Amphotericin B	2.29	0.72–7.28	0.15
Lack of source control	1.47	0.77–2.80	0.23
*Candida* species			
*C. albicans*	1.23	0.77–1.95	0.37
*C. tropicalis*	1.87	0.99–3.55	0.052
*C. glabrata*	0.74	0.48–1.16	0.19
*C. parapsilosis*	0.78	0.34–1.79	0.56
Cox proportional hazards model			
Candidemia of undetermined source	2.04	1.28–3.26	0.003
Pitt score	1.14	1.073–1.21	<0.0001
*Candida tropicalis*	2.08	1.084–4.013	0.028
Age (years)	1.02	1.0053–1.038	0.009
Neutropenia (previous 30 days)	3.28	1.69–6.33	<0.0001

## Data Availability

Supporting data is available from the corresponding author upon request.

## Supplementary Materials

The following are available online at https://www.mdpi.com/article/10.3390/jof8020123/s1, Table S1: Definitions used for sources of Candida bloodstream infection; Table S2: Clinical features of patients with urinary-origin Candida bloodstream infection.
