# Supplementary material for: Frequency and Clinical Features of Candida Bloodstream Infection Originating in the Urinary Tract
Source: J Fungi (Basel). 2022 Jan 27;8(2):123. doi: 10.3390/jof8020123 (PMC8878273; doi:10.3390/jof8020123)
Supplement: Supplementary file 1 [file jof-08-00123-s001.zip › jof-1555209-supplementary.pdf]

Table S1. Definitions used for sources of Candida bloodstream infection.

Source of CBSI	Definition
Urinary CBSI	Presence of at least 2 of 4 criteria, concomitant with the onset of candidemia:<br>1.Signs and symptoms of upper urinary tract infection (flank pain and tenderness);<br>2.Transurethral instrumentation;<br>3.Recovery of the same Candida species from blood and urine cultures obtained within 24 hours of the onset of candidemia<br>4.Radiological (US or CT) findings supporting UTI (abscess, evidence of pyelonephritis etc).<br>And no alternative source of Candida bloodstream infection identified.<br>**Probable urinary source**: ≥2 criteria, one of which is the microbiological criterion (criterion 3).<br>**Presumptive urinary source**: ≥2 criteria, criterion 3 is not met.
Gastrointestinal	Presence of at least 1 of 3 criteria, concomitant with the onset of candidemia:<br>1.Abdominal pain and/or diarrhea<br>2.Recent abdominal surgery<br>3.Imaging study demonstration GI pathology (enteritis, colitis, perforation, abscess, typhlitis etc.)<br>**And** no alternative source of Candida bloodstream infection identified
Hepatobiliary	Presence of at least 2 of 3 criteria, concomitant with the onset of candidemia:<br>1.Abdominal pain and/or tenderness<br>2.Abnormal cholestatic liver function test<br>3.Abdominal imaging (CT/US) demonstrating bile duct pathology or liver abscess or known bile duct instrumentation (eg. stent or PTD).<br>**And** no alternative source of Candida bloodstream infection identified
CVC associated CBSI	Candidemia that appears in the presence of a central venous catheter or within 48 h of removal of a central venous catheter and which cannot be attributed to an infection unrelated to the catheter.
Undetermined origin	Candidemia that does not meet the criteria for other sources as stated above.

Table S2. Clinical features of patients with urinary-origin Candida bloodstream infection.

No.	Age	Sex	Clinical details	Medical history	Urine culture	Blood culture	Imaging results
1	93	M	Pt. admitted from LTCF with fever, urinary retention and C-BSI on admission. Bladder catheter inserted on admission.	Dementia<br>BPH<br>HTN<br>Hyperlipidemia<br>Recurrent DVT	*C. albicans*	*C. albicans*	Not done
2	76	M	Pt. admitted from LTCF with fever, urinary retention and C-BSI on admission. Bladder catheter inserted on admission.	BPH<br>Dementia<br>DM<br>PVD<br>HTN<br>IHD<br>Hyperlipidemia	*C. tropicalis*	*C. tropicalis*	CT: Distended bladder with indwelling catheter. No evidence of hydronephrosis.
3	52	M	Fever and chills 4 days after PCNL.	DM<br>HTN<br>Hyperlipidemia<br>Nephrolithiasis	*C. albicans*	*C. albicans*	CT: Fat infiltration around right kidney. Enhancement of uroepithelium in right renal pelvis and ureter.
4	91	M	Pt. admitted from LTCF with fever, C-BSI on admission. Discharged from hospital 14 days earlier. Bladder catheter in previous hospitalization. *C. albicans* grew in urine and blood cultures. No alternative source of C-BSI identified.	Dementia<br>CVA<br>HTN<br>Hyperlipidemia<br>MDS	*C. albicans*	*C. albicans*	Not done
5	95	F	Pt. admitted with fever, C-BSI on admission, no alternative source of infection.	Dementia<br>DM<br>HTN<br>S/P DVT<br>Breast cancer	*C. glabrata*	*C. glabrata*	CT: Left renal pelvis markedly distended with sediment in the renal pelvis and bladder
6	88	M	Pt admitted with DKA, STEMI and acute kidney injury. Bladder catheter inserted on admission. On admission urine culture	Dementia<br>DM<br>HTN	*C. glabrata*	*C. glabrata*	CT: Multiple abscesses in both kidneys.

			positive for *C. tropicalis* and *C. glabrata*, blood culture negative, bladder catheter replaced. On HD-15 catheter replaced due to urinary retention. On HD-23 C-BSI with sepsis.	IHD<br>Nephrolithiasis<br>Hyperlipidemia			
7	74	M	Pt. with TCC, C-BSI on POD-9 after radical cystoprostatectomy.	TCC<br>S/P lobectomy d/t lung cancer	Few colonies of gram positive cocci	*C. albicans*	CT: Bladder mass with obstruction of left ureter
8	62	F	Pt. with ESRD. C-BSI 2 weeks after kidney transplantation. Leak of ureteral-bladder anastomosis.<br>*C. glabrata* grew in urine, blood and drain inserted into urinoma.	S/p breast cancer<br>Hyperlipidemia<br>ESRD	*C. glabrata*	*C. glabrata*	CT: Hematoma around transplanted kidney. Leak from anastomosis of right ureter to urinary bladder. Free fluid in the pelvis and abdomen.
9	83	M	Pt. admitted 30 days after radical cystectomy and ileal conduit, with fever and lower abdominal pain. C-BSI on admission.	Dementia<br>TCC<br>CLL<br>HTN<br>S/P Thyroid cancer<br>Hyperlipidemia	*C. glabrata*	*C. glabrata*	CT: Bilateral hydronephrosis due to extensive retroperitoneal lymphadenopathy. Suspected liver metastasis.
10	89	M	Pt admitted with urinary retention and hematuria.<br>C-BSI on admission.	Dementia<br>BPH<br>Epilepsy<br>CVA<br>HTN<br>Atrial fibrillation<br>Severe aortic stenosis	*C. glabrata*	*C. glabrata*	CT: Right nephrolithiasis without evidence of obstruction.
11	79	F	Pt. admitted with fever and altered mental status. Bladder catheter inserted on admission. She was treated empirically with Piperacillin-Tazobactam, with clinical improvement. Blood and urine cultures from admission were negative. C-BSI	Dementia<br>Hyperlipidemia<br>HTN<br>S/P breast cancer	*C. albicans*	*C. albicans*	US: no abnormal findings

			with fever and altered mental status on HD10.				
12	57	M	Pt. admitted with chills, dysuria and urinary retention. C-BSI on admission.	BPH<br>DM<br>Hyperlipidemia<br>Schizophrenia	*C. glabrata*	*C. glabrata*	US: Distended bladder, bilateral hydronephrosis. Hyperechoic sediment in bladder.<br>CT: contrast enhancement around left ureter and renal pelvis.
13	89	M	Pt. with pancreatic cancer and PTD. Admitted with abdominal pain and urinary retention. No evidence of PTD obstruction or cholangitis. Bladder catheter inserted on arrival to hospital. C-BSI on admission.	Pancreatic cancer<br>BPH<br>DM	*C. albicans*	*C. albicans*	Not done
14	86	M	Pt. with BPH and chronic indwelling bladder catheter. Admitted with fever and urinary retention. Catheter replaced on HD-1. C-BSI on admission.	Dementia<br>BPH<br>Gout<br>HTN<br>DM<br>Hyperlipidemia<br>PVD<br>CVA	*E. coli*	*C. glabrata*	Not done
15	82	M	Pt. admitted with fever, diarrhea and dysuria.<br>Urinary retention and bladder catheter insertion on admission. C-BSI on admission.	Dementia<br>IHD<br>Atrial fibrillation<br>HTN<br>DM<br>Hyperlipidemia<br>BPH	*C. glabrata*	*C. glabrata*	CT: Left perinephric fat infiltration
16	71	M	Pt with chronic indwelling bladder catheter. Admitted with abdominal pain and hematuria. C-BSI on admission.	Pancreatic cancer<br>DN<br>IHD<br>HTN<br>BPH	*C. tropicalis*	*C. tropicalis*	CT: Distended left kidney with perinephric fluid. Left hydronephrosis with 1.8 cm stone in renal pelvis. Gas in proximal left ureter. Suspected emphysematous pyelonephritis

				Essential thrombocytosis			
17	57	F	Pt with recurrent UTI admitted with 2 days of fever, chills, lower abdominal pain and left flank pain.	Hyperlipidemia<br>HTN	mixed bacterial growth	*C. glabrata*	CT: Distended left renal pelvis and ureter with enhancement of uroepithelium. Suspected SOL of proximal left ureter.
18	92	F	Pt. admitted from LTCF with sepsis and suspected right pneumonia. Blood and urine cultures from admission were negative. Bladder catheter inserted on HD-3, culture from catheter grew C. glabrata. C-BSI with fever and altered mental status on HD-6. Catheter was replaced.	Dementia<br>DM<br>HTN<br>IHD<br>Hyperlipidemia<br>Hypothyroidism	*C. glabrata* and *C. albicans*	*C. glabrata and C. albicans*	Not done
19	81	M	Admitted with seizure, intubated and sedated. Bladder catheter inserted on admission. C-BSI with sepsis on HD-28.	COPD<br>BPH<br>Hypothyroidism	*C. glabrata*	*C. glabrata*	Not done
20	90	M	Pt. admitted with bullous pemphigoid. Fever and urinary retention on HD-10. Bladder catheter inserted and cultures were taken. *C. glabrata* grew in urine and blood.	Dementia<br>Parkinson<br>CKD<br>HTN<br>Hyperlipidemia<br>Bullous pemphigoid	*C. glabrata*	*C. glabrata*	Not done
21	83	M	Pt. admitted after fall and hip fracture. Bladder catheter inserted in surgery. C-BSI on POD-19.	Dementia<br>IHD<br>DM<br>HTN<br>Atrial fibrillation<br>BPH<br>Hypothyroidism	*C. glabrata*	*C. glabrata*	US: Normal kidneys, without hydronephrosis. Prostate hypertrophy. Bladder wall is thickened and lobulated.
22	58	F	Pt. admitted for resection of pelvic tumor with portion of rectum and urinary bladder, protective colostomy and restoration of bladder with ileal conduit.	SCC of cervix<br>S/p breast cancer<br>DM<br>HTN	*C. albicans*	*C. glabrata* and *C. albicans*	CT: nephrostomy tubes and right ureter stent in place without hydronephrosis.

			Ureter stents were placed bilaterally. On POD-14 left ureteral stent was removed. A few hours later, C-BSI with fever and hemodynamic shock.				
23	65	F	Pt. admitted electively for tumor surgery. Bilateral ureter catheters inserted during operation. POD-6: C-BSI with fever, chills and flank tenderness.	Ovarian granulos cell tumor<br>Hypothyroidism<br>Hyperlipidemia	Not done	*C. albicans*	CT: bilateral nephrostomy tubes. Mild left hydronephrosis. Left kidney is distended, with cortical enhancement.
24	75	F	Pt. admitted with septic shock and C-BSI on admission.	Anal SCC<br>DM<br>Hyperlipidemia<br>s/p colon cancer	*C. glabrata*	*C. glabrata*	CT: severe bilateral hydroureteronephrosis.<br>Bladder wall with lobulated foci, consistent with abscesses versus progression of neoplasm.
25	54	F	Pt. admitted with fever and flank pain. C-BSI on admission.	Nephrolithiasis<br>DM<br>PBC	*C. glabrata*	*C. glabrata*	CT: 15 mm stone in right ureter
26	87	M	Pt. admitted with fever and dysuria. C-BSI on admission.	BPH<br>DM<br>HTN<br>Atrial fibrillation<br>Psoriasis	*E. coli*	*C. glabrata*	Not done
27	68	M	Pt. with TCC, 5 months after radical cystectomy with ileal conduit. Bilateral ureteral stents. Admitted with abdominal pain and acute kidney injury. On HD2 ureteral stents were exchanged with purulent discharge from ureters.<br>CBSI on HD-23.	TCC<br>HTN<br>Hyperlipidemia	mixed bacterial growth	*C. albicans*	CT: new onset bilateral hydronephrosis.
28	67	M	Pt. admitted electively for TCC resection. C-BSI with septic shock on POD-14 after radical cystectomy with ileal conduit.	TCC	*C. glabrata*	*C. glabrata*	Not done

Pt. : patient; LTCF: long term care facility; TCC: transitional cell carcinoma; SCC: squamous cell carcinoma; C-BSI: Candida bloodstream infection; POD: postoperative day; BPH: benign prostatic hypertrophy; HD: hospital day; PTD: percutaneous transhepatic drain; PCNL: percutaneous nephrolithonomy; CLL: chronic lymphocytic leukemia; UPJ: uretero-pelvic junction; SOL: space occupying lesion; STEMI: ST elevation myocardial infarction; DKA: diabetic ketoacidosis; DM: diabetes mellitus; HTN: hypertension; IHD: ischemic heart disease; CHF: congestive heart failure; CKD: chronic renal failure; DVT: deep vein thrombosis; PBC: primary biliary cirrhosis.
